# Strong Cross‐Reactivity of Anti–*Plasmodium falciparum* pLDH Monoclonal Antibodies With *Plasmodium malariae* pLDH in the Abbott Bioline Malaria Ag P.f/P.f/P.v Rapid Diagnostic Test

**DOI:** 10.1111/tmi.70142

**Published:** 2026-04-23

**Authors:** Gabriel Montoia, Maria Carmen Arroyo Sanchez, Giselle Lima‐Cooper, Maria de Lourdes Rego Neves Farinas, Juliana Inoue, Silvia Maria Di Santi

**Affiliations:** ^1^ Programa de Pós Graduação em Doenças Infecciosas e Saúde Global, Departamento de Infectologia e Medicina Tropical, Faculdade de Medicina Universidade de São Paulo São Paulo Brazil; ^2^ Laboratório de Soroepidemiologia, Instituto de Medicina Tropical, Faculdade de Medicina Universidade de São Paulo São Paulo Brazil; ^3^ Ryan White Center for Pediatric Infectious Disease and Global Health Indiana University School of Medicine Indianapolis Indiana USA; ^4^ Universidade Anhembi Morumbi Piracicaba Brazil; ^5^ Institute of Tropical Medicine, Eberhard Karls University of Tübingen Tübingen Germany; ^6^ Instituto Adolfo Lutz, Secretaria de Estado da Saúde de São Paulo São Paulo Brazil

**Keywords:** lactate dehydrogenase, malaria diagnosis, *Plasmodium falciparum*, *Plasmodium malariae*, rapid diagnostic test

## Abstract

**Objectives:**

This study aimed to describe the cross‐reactivity of the Abbott Bioline Malaria Ag P.f/P.f/P.v rapid diagnostic test, designed to detect *Plasmodium falciparum* and *P. vivax* , with other *Plasmodium* species, based on the detection of parasite lactate dehydrogenase enzymes.

**Methods:**

Blood samples from 40 patients with positive thick and thin blood smear results were analysed: 10 infected with *P. falciparum*, 10 with *P. vivax* , 15 with *P. malariae* and five with *P. ovale* . Parasitemia was quantified as parasites/μL of blood. All samples were tested using the Abbott Bioline Malaria Ag P.f/P.f/P.v RDT, and species identification was confirmed by nested PCR. To compare test line intensities and assess potential nonspecific reactions, images of the RDT lines were analysed using ImageJ software. For statistical analyses, PCR was considered the gold standard. Sensitivity, specificity and accuracy were calculated using MedCalc 2026. Fisher's exact test was applied using GraphPad/Quick Calcs to compare the specificity of the anti‐Pf‐pLDH antibodies, with a significance level of 0.05 (*p* < 0.05).

**Results:**

The RDT accurately detected all *P. falciparum* and *P. vivax* samples. However, 12 of 14 *P. malariae* samples yielded positive results on the T2 line, intended to be specific for Pf‐pLDH, as did four *P. ovale* isolates. The specificity of the anti‐Pf‐pLDH monoclonal antibodies against *P. malariae* was only 14.29% (*p* < 0.05). ImageJ analysis showed that the T2 line signal intensity in *P. malariae*‐infected samples exceeded that observed in *P. falciparum*–infected samples.

**Conclusions:**

This study provides the first evidence of cross‐reactivity of anti–Pf‐pLDH monoclonal antibodies in the Abbott Bioline Malaria Ag P.f/P.f/P.v RDT with *P. malariae* and *P. ovale* isolates. Moreover, highlights the imperative need to validate alternative biomarkers and novel point‐of‐care platforms, to enhance the rapid and accurate diagnosis of malaria, ensure species‐specific treatment and ultimately support effective malaria control and elimination strategies.

## 1 Introduction

Despite ongoing efforts to control and eliminate malaria, the disease leftovers a major global public health concern and has reached an epidemiological peak over the past two decades. In 2024, an estimated 282 million cases and 610,000 deaths were reported, with pregnant women and children under 5 years of age representing the most vulnerable groups [1]. Six *Plasmodium* species are known to infect humans: *P. falciparum*, *P. vivax* , *P. malariae*, *P. ovalecurtisi*, *P. ovalewallikeri* and *P. knowlesi* [2].

Although thick blood smear (TBS) remains the gold standard for malaria diagnosis, species differentiation can be difficult. The thin blood smear is a helpful technique for identifying characteristic forms and features that may not be visible in the TBS. Moreover, it requires minimal laboratory infrastructure and well‐trained personnel. Rapid diagnostic tests (RDTs) have emerged as a critical solution, revolutionising the way malaria is detected, treated and controlled, particularly in settings with limited access to microscopy. RDTs are immunochromatographic lateral flow devices that detect *Plasmodium* antigens within minutes, thereby enabling timely clinical decision‐making and reducing the risk of severe malaria. Furthermore, the use of RDTs when microscopy is unavailable helps to prevent the empirical administration of antimalarial drugs, contributing to the reduction of drug resistance [3]. The main biomarkers used in RDTs are *P. falciparum*‐specific histidine‐rich protein 2 (PfHRP2); *Plasmodium* lactate dehydrogenase specific for *P. falciparum* (Pf‐pLDH), specific for *P. vivax* (Pv‐pLDH), or pan‐malarial (pan‐pLDH) and *Plasmodium* aldolase, also a pan‐malarial antigen [4]. Molecular diagnosis offers the advantage of identifying and distinguishing *Plasmodium* species with high sensitivity and specificity, even at low parasitemia levels, with limits of detection (LoD) reaching 0.03 parasites/μL [5]. These methods are excellent tools for screening asymptomatic individuals in epidemiological surveys, for screening of blood, bone marrow and solid‐organ donors [6, 7, 8]. However, molecular protocols require laboratory infrastructure, trained personnel, are expensive and time‐consuming, which limits their use in routine clinical practice and in remote areas [9].

The WHO, in partnership with the Special Programme for Research and Training in Tropical Diseases (TDR), the Foundation for Innovative New Diagnostics (FIND), the Centers for Disease Control and Prevention (CDC) and other partners, conducts a rigorous quality‐assessment scheme for malaria RDTs. This assessment includes specificity testing for *P. falciparum* and *P. vivax* and cross‐reactivity assessments with other diseases, such as dengue, Chagas disease, leishmaniasis, schistosomiasis and syphilis [10].

In this study, we evaluated the cross‐reactivity of the Abbott Bioline Malaria Ag P.f/P.f/P.v test against other *Plasmodium* species, based on the detection of lactate dehydrogenase enzymes. The Abbott Bioline Malaria Ag P.f/P.f/P.v test contains a membrane strip pre‐coated with mouse monoclonal antibodies specific for *P. falciparum* HRP2 (test line T1), mouse monoclonal antibodies specific for Pf‐pLDH (test line T2) and mouse monoclonal antibodies specific for Pv‐pLDH (test line T3). As the blood samples containing *P. falciparum* and/or *P. vivax* antigens migrate along the membrane after the addition of the diluent, they form complexes with mouse monoclonal antibodies specific to *P. falciparum* HRP2 and pan‐pLDH conjugated to gold. These antigen–antibody complexes move through the membrane towards the test‐line regions, where they are captured by murine antibodies against *P. falciparum* HRP2, Pf‐pLDH and Pv‐pLDH, resulting in the appearance of a red‐purple band at the corresponding lines, indicating a positive reaction [11].

The present study provides the first report of a cross‐reactivity of the anti–Pf‐pLDH monoclonal antibodies in the Abbott Bioline Malaria Ag P.f/P.f/P.v RDT when applied to *P. malariae* and *P. ovale* isolates.

## 2 Methods

### 2.1 Study Design

According to the National Program for the Prevention, Control and Elimination of Malaria (PNPCEM) of the Brazilian Ministry of Health, diagnosis is performed using TBS and thin blood smears, as well as RDTs. This study was designed following the observation of disagreeing diagnostic results, in which patients attended at the Malaria Reference Laboratory, Adolfo Lutz Institute, Health Department of Sao Paulo State/Hospital das Clínicas, School of Medicine, University of Sao Paulo, and diagnosed with *P. malariae* or *P. ovale* by TBS and thin blood smears, showed positive results for Pf‐pLDH on RDTs. Based on these findings, samples were subsequently collected for further analysis, including molecular diagnosis.

Samples from 40 patients positive by TBS and thin blood smears were tested: 10 positives for *P. falciparum*, 10 for *P. vivax*, 15 for *P. malariae* and five for *P. ovale* . The parasitemia was calculated as the number of parasites/μL of blood, assuming 6000 WBC/μL for all patients [12]:

$$P=\frac{6,000\;\mathrm{WBC}\;x\;\text{number of parasites in}\;100\;\mathrm{WBC}}{100\;\mathrm{WBC}}$$

All samples were collected after informed consent. The *Plasmodium* species were confirmed by nested PCR for detection of *P. vivax* , *P. falciparum*, *P. malariae* and *P. ovale* [13].

### 2.2 Molecular Diagnosis

DNA extraction was carried out with QIAamp DNA Blood Mini Kit (QIAGEN, Hilden, Germany), according to the manufacturer's instructions. The DNA was eluted in a final volume of 50 μL and stored at −20°C for subsequent procedures.

Nested PCR was used to detect the *Plasmodium* species in the positive samples, using ssrRNA genes as target. The first reaction used genus‐specific primers rPLU5 and rPLU6 to amplify a 1.2 Kb fragment, and the nested reaction used species‐specific primers for *P. falciparum* (rFAL1/2), *P. vivax* (rVIV1/2), *P. malariae* (rMAL1/2) and *P. ovale* (rOVA1/2). The 25 μL reaction used 250 nM of each primer, 125 μM dNTPs, 2 mM MgCl_2_, 50 mM KCl, 10 mM Tris pH 8.3, 0.4 U of *Taq* polymerase and 1 μL of genomic DNA. Amplification was conducted at 95°C for 5 min; 58°C for 2 min; 72°C for 2 min; 24 cycles at 94°C for 1 min; 58°C for 2 min; 72°C for 2 min; final extension cycle at 72°C for 5 min. For the nested reaction, 30 amplification cycles were performed under the same conditions. The fragments obtained were separated by electrophoresis in 1.5% agarose gel in TBE buffer and visualised with GelRed (Biotium, Fremont, USA) through UV light with a molecular weight marker of 100 bp [13].

### 2.3 Rapid Diagnostic Tests

**FIGURE 1:**
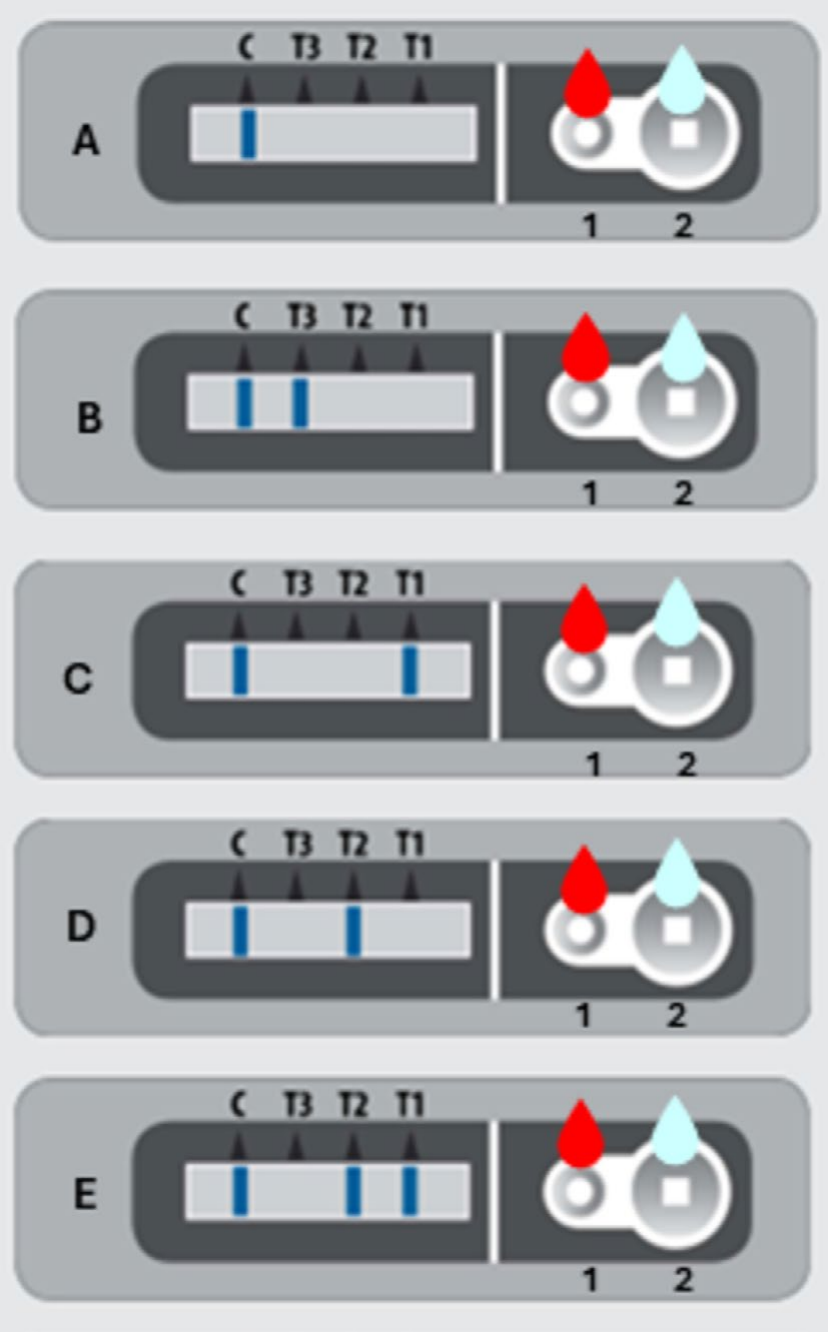
Schematic representation of Abbott Bioline Malaria Ag P.f/P.f/P.v RDT showing only the control line C in a negative result (A); *P. vivax* pLDH line (B); *P. falciparum* HRP2 line (C); Pf‐pLDH line (D); Pf HRP2 and pLDH lines (E). Font: Adapted from Brasil, 2013 [14].

The Abbott Bioline Malaria Ag P.f/P.f/P.v RDTs (05FK120‐25 T/Kit and 05FK123‐POCT) were applied to samples, according to the manufacturer instructions. In Brazil, the PNPCEM of the Ministry of Health distributes the Abbott Bioline Malaria Ag P.f/P.f/P.v to public services responsible for the care of malaria cases in endemic and non‐endemic areas [14]. In summary, 5 μL of blood were added to well 1, followed by four drops of diluent buffer in well 2 of the device, with the reading taken after 15 min (Figure 1). The following batches were tested: 05GDC004A, 05GDC006B, 05GDD001A, 05GDE004A, 05GDE004B, 05GDF001A, 05GDG002B, 05GDG003A, 05GDG003B, 05GDH005C, 05GDH005D, 05GDH005E, 05GDI002A, 05GDI002B, 05GDI010D, 05GDI011A, 05GDI011B, following the criteria adopted by the Malaria Reference Laboratory.

### 2.4 Measurement of Line Intensity of Rapid Diagnostic Tests

To compare line intensities independently of visual interpretation, enabling the correlation between signal intensity and parasitemia as well as the assessment of potential nonspecific reactions, images of the RDT lines were analysed using ImageJ software (version 1.53; National Institutes of Health, USA). Following visual reading of the RDTs, digital images of the devices were captured under standardised conditions of illumination, distance and focus to minimise interferences during image capture and subsequent software analysis. Images in.jpg format were imported into ImageJ and converted to 8‐bit grayscale, with pixel values ranging from 0 (absolute black) to 255 (absolute white). For each RDT, the central area of the reading window was selected, covering the positions of the control (C) and test lines (T1, T2 and T3). From the selected region, a grayscale density profile was generated using the Plot Profile function, which represents the variation in pixel intensity values along the longitudinal axis of the selection. Line intensity was determined as the difference between the baseline value of the nitrocellulose membrane and the minimum value of the curve corresponding to the C, T1, T2 and T3 lines. Signals clearly distinct from the baseline were considered positive [15].

### 2.5 Statistical Analyses

PCR was considered the gold standard test, and the results were analysed using statistical software. MedCalc (2026 MedCalc Software Ltd) was used to calculate the sensitivity, specificity and accuracy of the Abbott Bioline Malaria Ag P.f/P.f/P.v RDT, using anti‐Pv‐pLDH antibodies and anti‐Pf‐pLDH antibodies. GraphPad/Quick Calcs (GraphPad Software Inc., San Diego, CA, USA) was used to compare the specificity of the Abbott Bioline Malaria Ag P.f/P.f/P.v RDT, using anti‐Pf‐pLDH antibodies with Fisher's exact test. A significance level of 0.05 was considered (*p* < 0.05).

## 3 Results

### 3.1 Diagnosis According to Thick Blood Smear, Rapid Diagnostic Test and Nested PCR

Parasitemia as determined by ranged from 480 to > 100,000 parasites/μL, considering both asexual and sexual forms detected in peripheral blood. Of the 40 samples diagnosed by TBS and thin blood smears, 39 were confirmed by nested PCR, showing complete concordance between the methods and no evidence of mixed infection. One sample diagnosed by TBS and thin blood smear as *P. malariae* with rare parasites showed a negative result in the nested PCR and was therefore excluded from the study. Data from all samples are presented in Table 1.

**TABLE 1:** Description of *P. falciparum*, *P. vivax* , *P. malariae* and *P. ovale* isolates according to thick and thin blood smear, nested PCR, Abbott Bioline Malaria Ag P.f/P.f/P.v RDT and place of infection. *n* = 39.

Sample ID	TBS‐T	Parasitemia/μL	PCR	RDT T3/T2/T1	Place of infection
1F	Pf	86,820	Pf	−/+/+	PA/Brazil
2F	Pf	18,960	Pf	−/+/+	Sierra Leoa
3F	Pf	> 100,000	Pf	−/+/+	AP/Brazil
4F	Pf	8460	Pf	−/−/+	Nigeria
5F	Pf	22,020	Pf	−/+/+	Mozambique
6F	Pf	> 100,000	Pf	−/+/+	Angola
7F	Pf	18,600	Pf	−/+/+	Angola
8F	Pf	45,840	Pf	−/+/−	Angola
9F	Pf	73,080	Pf	−/+/+	Angola
10F	Pf	75,120	Pf	−/+/+	Ivory Coast
1V	Pv	31,920	Pv	+/−/−	NA
2V	Pv	14,760	Pv	+/−/−	RO/Brazil
3V	Pv	16,920	Pv	+/−/−	RO/Brazil
4V	Pv	41,760	Pv	+/−/−	RO/Brazil
5V	Pv	15,000	Pv	+/−/−	RO/Brazil
6V	Pv	14,820	Pv	+/−/−	RO/Brazil
7V	Pv	16,140	Pv	+/−/−	RO/Brazil
8V	Pv	NA	Pv	+/−/−	RO/Brazil
9V	Pv	1440	Pv	+/−/−	RO/Brazil
10V	Pv	1200	Pv	+/−/−	RO/Brazil
1M	Pm	25,140	Pm	−/+/−	SP/Brazil
3M	Pm	3780	Pm	−/+/−	Angola
4M	Pm	39,360	Pm	−/+/−	Angola
5M	Pm	1800	Pm	−/+/−	SP/Brazil
6M	Pm	2940	Pm	−/−/−	Angola
7M	Pm	18,600	Pm	−/+/−	Angola
8M	Pm	517,980	Pm	−/+/−	SP/Brazil
9M	Pm	6480	Pm	−/+/−	Angola
10M	Pm	6960	Pm	−/+/−	French Guiana
11M	Pm	2040	Pm	−/+/−	Cameroon
12M	Pm	7800	Pm	−/+/−	Nigeria
13M	Pm	8100	Pm	−/+/−	Angola
14M	Pm	7800	Pm	−/−/−	Nigeria
15M	Pm	480	Pm	−/+/−	SC/Brazil
1‐Ov	Po	540	Po	−/+/−	Tanzania
2‐Ov	Po	3480	Po	−/+/−	Nigeria
3‐Ov	Po	12,720	Po	−/+/−	Africa^a^
4‐Ov	Po	3900	Po	−/+/−	Africa^a^
5‐Ov	Po	600	Po	−/−/−	Africa^a^

Abbreviations: NA, not available; Pm, *P. malariae*; Po, *P. ovale* ; Pv, *P. vivax* ; RDT, rapid diagnostic test; Pf, *P. falciparum*; T1, histidine‐rich protein 2 of *P. falciparum*; T2, lactate dehydrogenase of *P. falciparum*; T3, lactate dehydrogenase of *P. vivax* ; TBS‐T, thick and thin blood smear.

^a^ Country not available.

**FIGURE 2:**
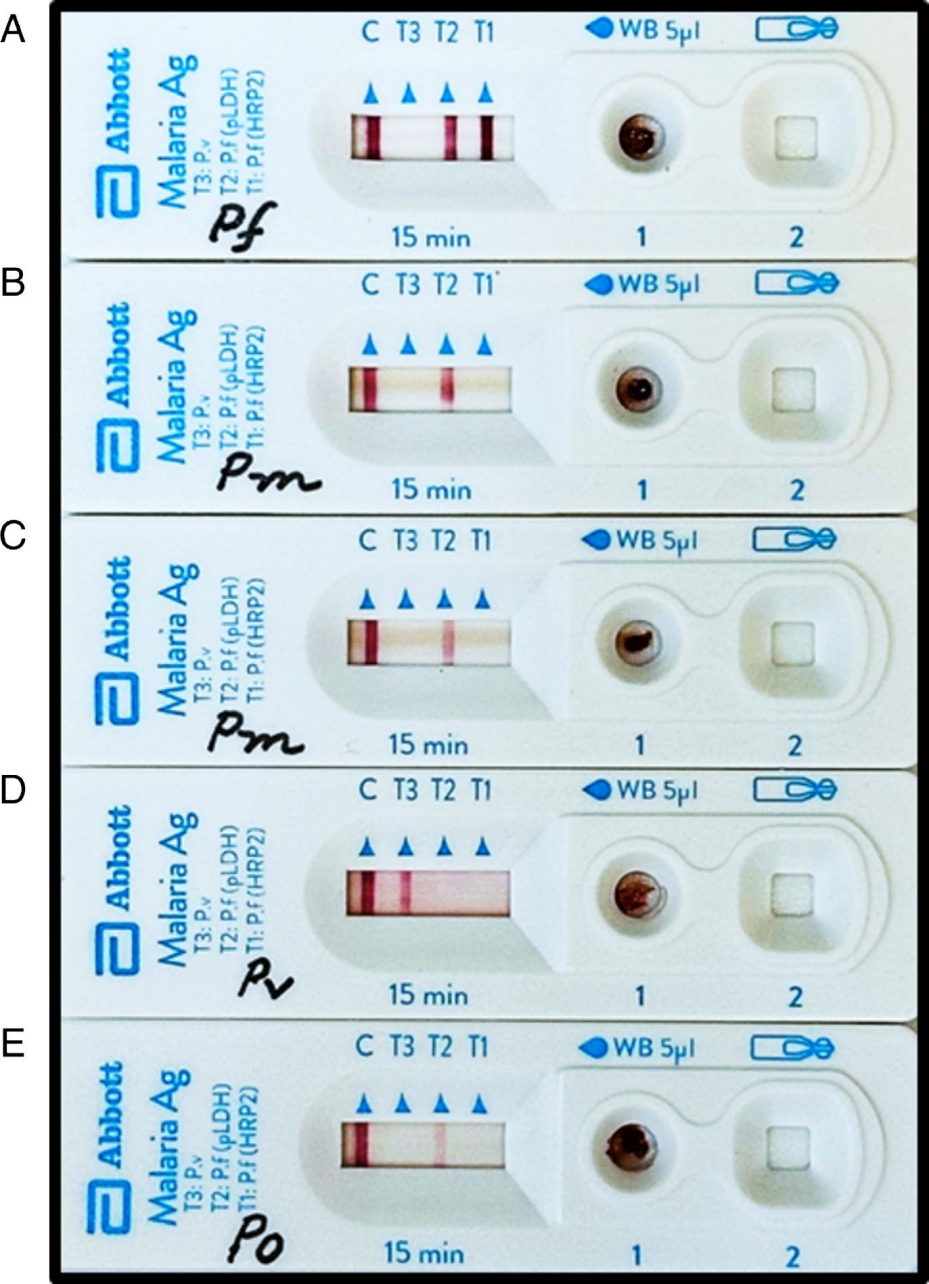
Results of Abbott Bioline Malaria Ag P.f/P.f/P.v RDT tested in *P. falciparum* (A), *P. malariae* (B,C), *P. vivax* (D) and *P.ovale* (E). Cross reactivity of antibodies anti‐Pf‐pLDH with *P. malariae* and *P. ovale* antigens are shown in, C and E devices, presenting visible lines 2. C=Control line; T1 = PfHRP2 line; T2 = Pf‐LDH line; T3 = Pv‐LDH line.

RDTs were positive for all 10 *P. falciparum* isolates: eight samples were positive for both T1 and T2 lines, one was positive exclusively for T1 and one exclusively for T2. Regarding *P. vivax* , all samples yielded positive results in the T3 line. Unexpectedly, 12 of 14 *P. malariae* samples showed a positive result in the T2 line, as did four of five *P. ovale* isolates, as illustrated in Figure 2.

Statistical analyses demonstrated a sensitivity of 100% for anti–Pv‐pLDH antibodies in detecting *P. vivax* antigens, as well as 100% specificity against *P. falciparum* and *P. malariae* antigens. In contrast, anti–Pf‐pLDH antibodies showed a sensitivity of 90% for *P. falciparum* antigens, lower than the 97.4% reported by the manufacturer. The specificity of these monoclonal antibodies against *P. malariae* antigens was only 14.29%, highlighting the limited ability of these RDTs to exclusively detect Pf‐LDH (Table 2).

**TABLE 2:** Sensitivity, specificity and accuracy of the Abbott Bioline Malaria Ag P.f/P.f/P.v RDT, showing a cross reactivity of anti Pf‐pLDH antibodies with *P. malariae* antigens.

anti Pv‐pLDH antibodies (T3 line)								
PCR	*n*	TP	FN	TN	FP	Statistic	Value	95% CI
*Pv*	10	10	0			Sensitivity	100.00%	69.15% to 100.00%
*Pf*	10			10	0	Specificity	100.00%	69.15% to 100.00%
*Pm*	14			14	0	Specificity	100.00%	76.84% to 100.00%
no *Pv* (*Pf* + *Pm*)	24			24	0	Specificity	100.00%	97.75% to 100.00%
						Accuracy	100.00%	89.72% to 100.00%
anti Pf‐pLDH antibodies (T2 line)								
PCR	n	TP	FN	TN	FP	Statistic	Value	95% CI
*Pf*	10	9	1			Sensitivity	90.00%	55.50% to 99.75%
*Pv*	10			10	0	Specificity	100.00% *	69.15% to 100.00%
*Pm*	14			2	12	Specificity	14.29%*	1.78% to 42.81%
no *Pf* (*Pv* + *Pm*)	24			12	12	Specificity	50.00%	29.12% to 70.88%
						Accuracy	61.76%	43.56% to 77.83%

*Note:* Grey shading areas = not applicable.

Abbreviations: FN, false negative; FP, false positive; *n*, number; Pf‐pLDH, lactate dehydrogenase of *P. falciparum*; Pv‐pLDH, lactate dehydrogenase of *P. vivax* ; RDT, rapid diagnostic test; TN, true negative; TP, true positive.

* Fisher's exact test, *p* < 0.0001.

The analysis of T2 line intensity performed using ImageJ software [15] revealed a relevant finding of nonspecific reactivity in the Abbott Bioline Malaria Ag P.f/P.f/P.v. The T2 line signal intensity observed in a *P. falciparum*–infected sample (3F) with parasitemia of > 100,000 parasites/μL was lower than that observed in a *P. malariae*–infected sample (10 M) presenting a substantially lower parasitemia (6960 parasites/μL).

**FIGURE 3:**
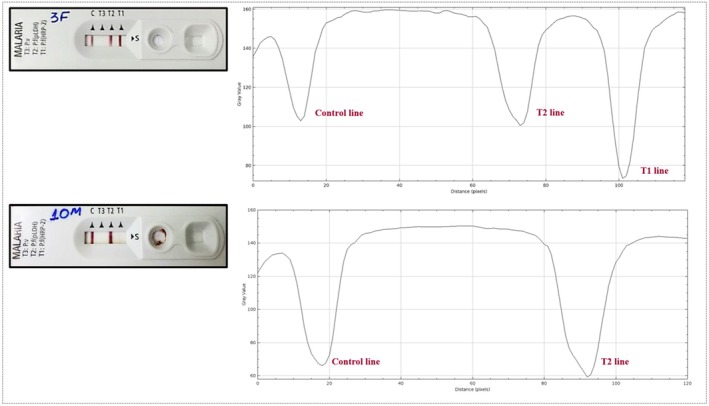
Analysis of lines intensities using ImageJ software showing the T2 line signal intensity in a *P. falciparum*–infected sample (3F) with parasitemia of > 100,000 parasites/μL, lower than that observed in a *P. malariae*–infected sample (10 M) presenting a substantially lower parasitemia (6960 parasites/μL) in the Bioline Malaria Ag P.f/P.f/P.v RDT.

In the grayscale density profile, the T2 line of sample 3F exhibited a moderate reduction in grayscale values relative to the baseline of the nitrocellulose membrane, consistent with a positive signal of limited intensity. In contrast, the *P. malariae* sample 10 M showed a deeper and broader decrease in the density profile corresponding to the T2 line, indicating a more intense colorimetric signal despite the markedly lower parasitemia (Figure 3).

## 4 Discussion

Accurate diagnosis and prompt malaria treatment are essential not only for reducing morbidity and mortality but also for preventing transmission and the emergence of drug‐resistant malaria parasites. Traditionally, diagnosis has relied on clinical symptoms and microscopy. However, these methods have limitations, particularly in resource‐limited settings; therefore, the need for efficient, accurate and accessible diagnostic methods, such as RDTs, is paramount [9].

The WHO/TDR/FIND/CDC Program evaluates RDTs against cryopreserved *P. falciparum* and *P. vivax* clinical samples from different regions, diluted to 200 and 2000 parasites/μL with consistently comparable concentration ranges of PfHRP2, pLDH and aldolase determined by ELISA, indicating which products are likely to be more sensitive in the field. According to this program, the Abbott Bioline Malaria Ag P.f/P.f/P.v RDT achieved a sensitivity of 89% for *P. falciparum* and 97.1% for *P. vivax* in the Panel Detection Score, with a parasitemia of 200 parasites/μL and 100% for both species with 2000 parasites/μL [10, 11]. The former name of the Abbott Bioline Malaria Ag P.f/P.f/P.v was SD Bioline Malaria Ag P.f/P.f/P.v [10].

Barney et al. [16], using recombinant and native pLDH proteins specific to *P. falciparum*, *P. vivax* , *P. malariae* and *P. ovale curtisi*, demonstrated in vitro cross‐reactivity between *P. ovale* and *P. malariae*‐specific pLDH proteins and anti Pf‐pLDH antibodies, suggesting an overlap of antigenic epitopes among pLDH proteins expressed by *P. falciparum*, *P. malariae* and *P. ovale* . The authors highlighted the need to further investigate the extent of cross‐reactivity in commercial RDTs, which is particularly relevant given that Pf‐pLDH has become one of the most important biomarkers, mainly due to the increase in reports of *hrp2/hrp3* deletions in *P. falciparum* isolates.

To the best of our knowledge, this study reports for the first time cross‐reactivity between monoclonal antibodies targeting Pf‐pLDH and *P. malariae* pLDH antigens in the commercial Abbott Bioline Malaria Ag P.f/P.f/P.v RDT. According to the manufacturer, this assay is intended for use in regions where *P. vivax* and *P. falciparum* are prevalent, in areas with reported *hrp2/hrp3* gene deletions and is listed as a WHO‐prequalified rapid diagnostic test. Although only a limited number of *P. ovale* isolates were tested, which constitutes a limitation of this study, similar results were observed for this species.

Our results showed no cross‐reactivity between antibodies targeting Pv‐pLDH and other antigens from other *Plasmodium* species, including *P. knowlesi* . This finding contrasts with the in vitro cross‐reactivity reported by Barney et al. [16] between antibodies against Pv‐pLDH and pLDH antigens from *P. knowlesi* and *P. cynomolgi*. These differences highlight the importance of evaluating commercial RDTs under routine diagnostic conditions, rather than relying exclusively on in vitro assays. The results obtained with the Abbott Bioline Malaria Ag P.f/P.f/P.v devices used in the routine of diagnosis were corroborated with the results from the ImageJ, where the T2 line intensity observed in *P. malariae* samples reflects cross‐reactivity, thereby characterising the lack of specificity of the evaluated RDT. The presence of a strong T2 signal in *P. malariae*–infected samples indicates that the monoclonal antibody employed in the T2 line is not exclusive to *P. falciparum* detection and may cross‐react also with antigens expressed by *P. ovale* . Such cross‐reactivity compromises the test's ability to accurately discriminate between species, particularly in settings of coinfection or in endemic areas where multiple *Plasmodium* species co‐circulate. Furthermore, because malaria treatment relies on accurate species‐level diagnosis, patients infected with *P. malariae* who are nonspecifically diagnosed as having *P. falciparum* infection may receive artemisinin‐based combination therapies (ACTs), whereas they could be effectively treated with chloroquine monotherapy. This misclassification may unnecessarily increase the use of artemisinin derivatives, thereby contributing to drug pressure and potentially favouring the emergence and spread of antimalarial resistance [9]. With respect to *P. ovale* , misdiagnosis as *P. falciparum* may result in inappropriate treatment with ACTs without the administration of primaquine or tafenoquine to target hypnozoites, leading to relapses and increased transmission due to the persistence of gametocytes in peripheral blood [17]. Furthermore, epidemiological data may be biased, including the mapping of *hrp2*/*3* gene deletions, as the presence of the T2 line alone is suggestive of gene absence [18].

Lateral flow devices represent an important tool in settings where microscopy is not available, particularly assays capable of separately detecting pLDH and PfHRP2 from *P. falciparum* isolates, thereby allowing parasite detection even in cases of *hrp2*/*3* deletions. The Abbott Bioline Malaria Ag P.f/P.f/P.v RDT has been among the best‐evaluated assays, with reported sensitivities of 94% for Pf‐pLDH at parasitemia levels > 200 parasites/μL and 98% for PfHRP2 [18]. However, recent reports have shown a sensitivity of only 45% for *P. falciparum* and 74% for *P. vivax* when using the Abbott Bioline P.f./P.v. in clinical samples with parasitemia > 200 parasites/μL. Notably, the geometric mean parasite density among 11 *P. falciparum* infections that tested negative by the Bioline RDT was 3488 parasites/μL, well above the minimal threshold of 200 parasites/μL recommended by WHO [19].

This study demonstrates, for the first time, cross‐reactivity between monoclonal anti–PfLDH antibodies used in the Abbott Bioline Malaria Ag P.f/P.f/P.v RDT with *P. malariae* and *P. ovale* isolates. These findings are particularly concerning, as they have the potential to jeopardise clinical decision‐making, epidemiological surveillance and malaria control and elimination strategies. This concern is especially relevant in endemic settings where microscopy is unavailable and RDTs represent the sole diagnostic tool.

There is therefore an urgent need for the identification of alternative biomarkers, as well as the development of novel point‐of‐care diagnostic platforms, to improve the prompt and accurate diagnosis of malaria, ensure appropriate species‐specific treatment and ultimately contribute to effective disease control and elimination efforts.

## 5 Conclusion

Rapid diagnostic tests constitute a crucial tool for malaria diagnosis in remote and resource‐limited settings, where most of the transmission cases occur. These assays are primarily based on the detection of PfHRP2, Pf‐pLDH and Pv‐pLDH biomarkers. Accurate diagnosis and prompt malaria treatment are fundamental pillars of malaria control and elimination, as they prevent onward transmission and limit the emergence of drug‐resistant parasites. This study reports, for the first time, cross‐reactivity between monoclonal antibodies targeting Pf‐pLDH used in the Abbott Bioline Malaria Ag P.f/P.f/P.v RDT and *P. malariae* pLDH, with 85.71% of the clinical samples yielding false‐positive results for T2 line, which is intended to be specific for *P. falciparum* detection. Additionally, anti Pf‐pLDH antibodies exhibited cross‐reactivity with *P. ovale* pLDH. The findings obtained with the Abbott Bioline Malaria Ag P.f/P.f/P.v devices were corroborated by the quantitative image analysis using ImageJ software, revealing a lack of specificity of the evaluated RDT. These results substantially compromise the test's ability to accurately discriminate between *Plasmodium* species and have important clinical and public health implications. Misclassification directly affects species‐specific malaria treatment, may lead to unnecessary use of artemisinin derivatives, thereby increasing drug pressure and potentially favouring the emergence and spread of antimalarial resistance, and raises the risk of relapses due to the absence of antimalarials targeting hypnozoites in *P. ovale* infections. Moreover, such diagnostic inaccuracies may bias epidemiological data, including surveillance efforts aimed at mapping *hrp2*/*3* gene deletions. These results are especially critical in endemic settings where RDTs represent the sole available diagnostic tool.

Overall, this study underscores the urgent need to identify and validate alternative biomarkers, as well as to develop novel point‐of‐care diagnostic platforms, to enhance the rapid and accurate diagnosis of malaria, ensure appropriate species‐specific treatment and ultimately support effective malaria control and elimination strategies.

## Funding

This work was supported by grants #2011/07380‐8 and #2012/18014‐5, São Paulo Research Foundation (FAPESP); Adolfo Lutz Institute; Fundo Especial de Saúde para Imunização em Massa e Controle de Doenças—FESIMA, Proc. SEI 024.00069348/2025‐09. GM was supported by a fellowship of Coordenação de Aperfeiçoamento de Pessoal de Nível Superior—Brasil (CAPES), Proc. 88887.183018/2025‐00. L‐CG was supported by a fellowship of Conselho Nacional de Desenvolvimento Científico e Tecnológico—Brasil (CNPq), Proc. 158023/2011‐0. MLF was supported by #2018/07890‐5, São Paulo Research Foundation (FAPESP); JI was supported by a fellowship of Coordenação de Aperfeiçoamento de Pessoal de Nível Superior—Brasil (CAPES). The Article Processing Charge for the publication of this research was funded by the Coordination for the Improvement of Higher Education Personnel (CAPES) (ROR identifier: 00x0ma614). For the purposes of open access, the authors have applied a Creative Commons CC BY licence to any accepted version of the article.

## Ethics Statement

This study was approved by the Ethics Committee of the Hospital das Clínicas of the School of Medicine of the University of Sao Paulo (CAAE: 66377317.2.0000.0068 and CAAE: 00770912.7.1001.0065).

## Conflicts of Interest

The authors declare no conflicts of interest.

## Data Availability

The data that support the findings of this study are available from the corresponding author upon reasonable request.
